# Peptidoglycan in osteoarthritis synovial tissue is associated with joint inflammation

**DOI:** 10.21203/rs.3.rs-2842385/v1

**Published:** 2023-04-28

**Authors:** Meaghan N Holub, Amanda Wahhab, Joseph R Rouse, Rebecca Danner, Mecaila M McClune, Jules M Dressler, Klemen Strle, Brandon L Jutras, Adam I Edelstein, Robert B Lochhead

**Affiliations:** Medical College of Wisconsin; Medical College of Wisconsin; Medical College of Wisconsin; Medical College of Wisconsin; Virginia Tech; Virginia Tech; Tufts University; Virginia Tech; Northwestern University; Medical College of Wisconsin

**Keywords:** osteoarthritis, peptidoglycan, synovitis, inflammation

## Abstract

**Objectives:**

Peptidoglycan (PG) is an arthritogenic bacterial cell wall component whose role in human osteoarthritis is poorly understood. The purpose of this study was to determine if PG is present in synovial tissue of osteoarthritis patients at the time of primary total knee arthroplasty (TKA), and if its presence is associated with inflammation and patient reported outcomes.

**Methods:**

Intraoperative synovial tissue and synovial fluid samples were obtained from 56 patients undergoing primary TKA, none of whom had history of infection. PG in synovial tissue was detected by immunohistochemistry (IHC). Synovial tissue inflammation and fibrosis were assessed by histopathology and synovial fluid cytokine quantification. Primary human fibroblasts isolated from arthritis synovial tissue were stimulated with PG to determine inflammatory cytokine response.

**Results:**

A total of 33/56 (59%) of primary TKA synovial tissue samples were positive for PG by IHC, with mean 8 PG occurrences per 10 mm^2^ of tissue in PG-positive samples. Synovial tissue inflammation and elevated IL-6 in synovial fluid positively correlated with PG positivity. Primary human fibroblasts stimulated with PG secreted high levels of IL-6, consistent with *ex vivo* findings. Interestingly, we observed a significant inverse correlation between PG and age at time of TKA, indicating younger age at time of TKA was associated with higher PG levels.

**Conclusion:**

Peptidoglycan is commonly found in synovial tissue from patients undergoing TKA. Our data indicate that PG may play an important role in inflammatory synovitis, particularly in patients who undergo TKA at a relatively younger age.

## Introduction

Osteoarthritis (OA) of the knee affects over one-third of the United States population aged 60 years or greater (1). The incidence of knee OA is expected to increase over the coming decades owing to the aging population and increases in obesity (2). Pain and functional limitations from knee OA have major impacts on quality of life for persons living with arthritis (3), and surgical management of lower extremity arthritis now comprises the largest procedural expenditure in the Medicare budget (4). Knee replacement surgery is associated with improvements in pain and function (5), but 15–20% of patients have continued pain and dissatisfaction following surgery (6, 7).

Osteoarthritis is characterized macroscopically by loss of articular cartilage and changes to subchondral bone, and involves changes to all tissues in the joint through a complex interplay of inflammatory molecules (8, 9). Synovial inflammation and hypertrophy occur in joints affected by OA (10, 11), and severity of synovitis positively correlates with symptoms (12, 13). Synovial inflammation present in OA is mediated by inflammatory cytokines (14), but disease mechanisms of inflammatory OA are incompletely understood. Owing to the growing disease burden of knee OA and the cost and imperfect outcomes associated with its treatment, there remains a critical need to better understand the pathophysiology of synovial inflammation both before and after knee replacement.

Microbial products derived from the microbiome have been proposed to contribute to joint inflammation in OA (15), and there is increasing evidence that circulating microbial debris contributes to OA (16). Peptidoglycan (PG), a structural component of the bacterial cell wall and a highly conserved pathogen-associated molecular pattern (PAMP), has been shown to trigger inflammatory responses in both Lyme arthritis (LA) and rheumatoid arthritis (RA) (17–19). PG is recognized by innate immune cells via pattern recognition receptors(18, 20, 21). Bacterial DNA and bacterial debris, including PG, have also been reported in synovium of limited cohorts of patients with OA (22–24), but the prevalence and potential impact of PG in synovium in patients with advanced knee arthritis remain incompletely understood.

The aim of this study was to characterize the prevalence of PG in synovial fluid and tissue samples at time of total knee arthroplasty performed for advanced OA, and to define its association with synovitis, inflammatory cytokines, and patient outcomes.

## Methods

### Patients

The present study was approved by the Medical College of Wisconsin and Froedtert Hospital Institutional Review Board (IRB) for Human Subject Research (PRO00035381, “Arthritis research at MCW”). Written informed consent was obtained from 66 patients undergoing elective, primary TKA with one of the senior authors (AE). None of the patients had a prior history of knee infection, prior knee surgery, or had an intra-articular injection within 3 months of surgery. We also enrolled 4 patients undergoing debridement and component explant due to periprosthetic joint infections to serve as positive controls. Ten of the OA patient samples were excluded due to excessive, nonspecific background staining. Of the remaining 56 quality samples, 53 patients had been diagnosed with degenerative arthritis.

### Clinical evaluation

Patient demographics and comorbidities were collected during the pre-operative appointment. Knee injury and Osteoarthritis Outcome Score for Joint Replacement (KOOS JR) and Veterans Rand-12 Health Survey (VR-12) scores were collected at baseline and again at 3, 6, and 12 months postoperatively. All patients were followed clinically for at least one-year post-operatively to monitor recovery and occurrences of complications. Pain and functional recovery were assessed by patient reported outcome measures; occurrence of any infectious complications or reoperations were recorded.

### Specimen collection

At the beginning of each patient’s TKA procedure, synovial fluid was aspirated from the operative knee using an 18-gauge needle following sterile prepping and draping and skin incision but prior to arthrotomy. Following arthrotomy, synovial tissue was harvested from the suprapatellar pouch and the medial and lateral gutters. Specimen was stored in sterile specimen containers and prepared for various analyses within 6 hours of collection.

### Specimen preparation and isolation

All synovial fluid and synovial tissue samples were collected and processed under sterile conditions and stored in −80° C freezer or liquid nitrogen until further use. If available, synovial fluid was flash-frozen and stored for downstream cytokine analysis. Synovium was isolated from collected synovial tissue using sterile surgical scissors and forceps and sectioned into small (1–2 mm^3^) tissue fragments. Two sections from each patient were embedded within optimal cutting temperature compound. Samples were stored in a −80° C freezer overnight, then transferred to liquid nitrogen for long term storage prior to histopathologic analysis.

### Histopathology

Two sections from each patient were used to assess inflammation by hematoxylin and eosin (H&E) stain and fibrosis by Masson’s trichrome stain. H&E-stained sections were qualitatively evaluated and blindly scored for markers of inflammatory synovitis on a scale of 0 to 3, with 3 being most severe. Three separate scores for overall inflammatory infiltrate, number of inflammatory foci, and synovial lining thickness were summed together to produce an overall inflammatory synovitis score for each patient sample. Each trichrome-stained section was scored in a blinded fashion using a scale of 0 to 3, with 3 being most severely fibrotic. All scores were independently reviewed prior to unblinding of the coded samples.

### Anti-peptidoglycan antibody generation

*B. burgdorferi* B31-A3 was cultured in complete BSK-II media supplemented with 6% rabbit serum. *Escherichia coli* strain K12; *Bacillus subtilis* strain 168; and *Staphylococcus aureus* (FDA 209); were propagated in Lysogeny Broth (LB), *Streptococcus mutans* strain Clark in Brain Heart Infusion (BHI) broth, and *Deinococcous radiodurans* strain 13939 in Tryptone Yeast (TY) media supplemented with 10% glucose.

All bacteria were grown to mid-exponential phase, harvested at 4,000 x g for 15 minutes, and then washed twice with PBS. For peptidoglycan purification, bacterial pellets were resuspended in PBS and added dropwise into boiling SDS (5% w/v, final concentration) and boiled for 1 hour as previously described(17). All Gram-positive bacteria were bead-beat (BeadBug, Benchmark Scientific) prior to SDS boiling for 3 cycles of 60 seconds on, 60 seconds on ice. After boiling, all samples were cooled to 30°C, and the pellets washed with autoclaved H_2_O four times using ultracentrifugation at 283,346 x g for 60 min at 30°C. The pellets were then resuspended in H_2_O and treated with lipase (1 mg/ml) for 3 hours, benzonase nuclease (4 µl/ml) for 2 hours, and overnight with chymotrypsin (0.3 mg/ml), all with shaking at 37°C. The next day 0.5% SDS was added to each pellet and heated to 80°C for 30 min. The pellets were washed 3 times with autoclaved H_2_O at the same centrifugation conditions listed above. The Gram-positive samples were treated with a final concentration of 1M HCl while continuously rotating at 4°C for 48 hours and centrifuged/washed 3 times, as described above. The dry weight was measured to quantify the amount of PG purified. To create the anti-peptidoglycan antibody, 5 BALB/cJ mice purchased from Jackson Laboratories were injected subcutaneously with 200 µg total of peptidoglycan from the bacteria listed above and mixed with equal parts of Freund’s Complete adjuvant (Thermo Scientific Ref: 77140) (2 mg/ml final of PG). After 3 weeks all mice received a 265 µg booster injection of the same PG mixture. 2.5 weeks later the mice were euthanized, and blood was collected. The blood was incubated at room temperature for 30 minutes prior to spinning at 1,500 x g for 10 min at 4°C. The serum was then removed, pooled together, and frozen at −20°C. The specificity of the antibody was tested using immunofluorescence and was found to bind *S. mutans, D. radiodurans*, *S. aureus*, and *E. coli* PG (data not shown) using methods described elsewhere (25, 26).

### PG staining and scoring:

Two sections of tissue from each patient were coded and stained by immunohistochemistry using the mouse anti-PG antiserum to label PG in synovial tissue, followed by incubation with horseradish peroxidase-conjugated goat anti-mouse IgG (Sigma-Aldrich) as detection antibody. Non-immunized mouse serum was used as a negative control. Following staining optimization for the custom anti-serum, all sections were processed at one time by staff at our core facility to control for technical variability. Slides that had nonspecific edge staining artifacts were excluded from further analysis. For each section (2 per patient), five 1mm^2^ fields were randomly selected from the tissue section and number of stained foci, corresponding to individual PG occurrences, were counted and summed across both sections (10 mm^2^ total area analyzed per patient sample). Samples were then scored from 0–4 based on the number of PG occurrences in tissue: 0 = no PG occurrences; 1 = 1–9 PG occurrences; 2 = 10–19 PG occurrences; 3 = 20–29 PG occurrences; 4 = 30 + PG occurrences.

### Primary human fibroblast isolation and stimulation

Fibroblasts were isolated from the human synovial tissue samples described above. A portion of the tissue fragments were transferred to a 15 ml conical centrifuge tube containing 5 ml of collagenase D (Sigma Aldrich 11088858001) at a concentration of 1 mg/ml (dissolved in Hank’s balanced salt solution (HBSS) [Sigma Aldrich 55037C] + 1% Penicillin/Streptomycin (Pen/Strep) [Fisher Scientific 15140122]). The tube was kept in a 37°C bead bath for 1 hour and was shaken vigorously every 5 minutes to release cells. Large tissue fragments were removed using sterile forceps and discarded. Remaining liquid was centrifuged at 1100 rpm for 10 minutes at room temperature. Supernatant was discarded and cell pellet was resuspended in 5 ml of enriched human fibroblast medium (High glucose DMEM [Sigma Aldrich D5671] + 20% fetal bovine serum (FBS) [BioWest S1690] + 1% Pen/Strep + 1% glutamine [Fisher Scientific 35050061] + 1% non-essential amino acids (NEAA) [Fisher Scientific 11140050] + 5 ng/ml recombinant human FGF-basic [BioLegend 792504]). Cells were then transferred to a T25 tissue culture flask and placed in a 37°C incubator with 5% CO2. Cell culture medium was replaced every 3–4 days, and cells were passaged at ~ 90% confluency. Primary fibroblasts were frozen at passage 4 and stored in liquid nitrogen.

### Fibroblast stimulation

Samples were passaged at least 6 times prior to use. Cells were plated in 24-well plates at 2.5 × 10^5 cells per well in 500 µl of medium. Each patient sample was plated in two wells, and one of the wells was stimulated with 10 µg/ml of the muramyl dipeptide fragment from *Staphylococcus aureus* peptidoglycan (Sigma Aldrich 77140) for 24 hours. Cell culture supernatants were collected and stored at −80° C until further analysis.

### Cytokine analysis

Cytokine analysis was performed using the LEGENDplex Human Inflammation Panel (Biolegend) to quantify 13 human inflammatory cytokines/chemokines (IL-1β, IFN-α2, IFN-γ, TNF-α, MCP-1 (CCL2), IL-6, IL-8 (CXCL8), IL-10, IL-12p70, IL-17A, IL-18, IL-23, and IL-33). Bead populations conjugated with antibodies specific to the mentioned cytokines/chemokines were incubated with neat synovial fluid samples allowing the target analytes to bind to the specific capture bead. Biotinylated detection antibodies were then combined with the analyte bound beads and each detection antibody formed a bond with their specific analyte. Thereafter, Streptavidin-phycoerythrin (SA-PE) was added to bind to the biotinylated detection antibodies generating a fluorescent signal with an intensity proportionate to amount of the specific cytokine/chemokine bound to the capture bead. Each sample was run through a flow cytometer where SA-PE fluorescence intensity was converted to cytokine/chemokine concentration based on a standard concentration curve.

### Statistical analysis

Statistical associations between PG severity scores and synovial inflammation, accumulation of fibrotic tissue, cytokine levels, population demographics, and patient reported outcome scores were assessed using Pearson correlations and regression analysis (p value cutoff = 0.05). Statistically significant differences in cytokine secretion levels between stimulated vs. unstimulated fibroblasts were determined by paired two-tailed t test (p value cutoff = 0.05). All statistical analyses were performed using GraphPad Prism (v.9).

## Results

### Patient characteristics and outcomes

In total, 56 samples from patients undergoing primary, elective TKA met our staining quality and inclusion criteria. The average age and BMI of our patient cohort was 67 years and 31.5 kg/m^2^, respectively (Table 1). Post-operative patient outcomes were measured using KOOS JR and VR-12 scores (Table 1). As expected, there was significant improvement in scores from baseline to final follow up. There were no occurrences of periprosthetic joint infection throughout the follow up period for the elective TKA cohort, and no patients underwent revision surgery.

### Identification of bacterial peptidoglycan within in synovial tissue

**Figure 1:**
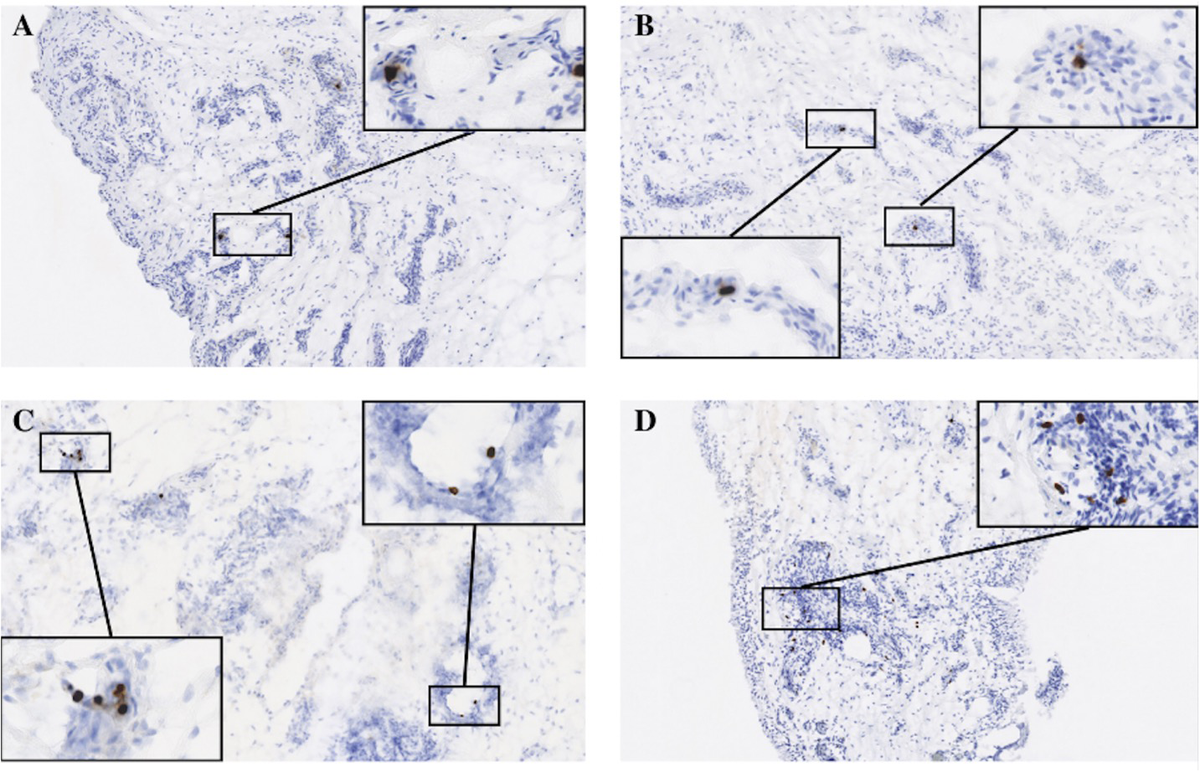
Detection of bacterial peptidoglycan (PG) in synovial tissue. Shown are representative sections of synovial tissue stained for PG by immunohistochemistry (see methods for details) obtained from (A) a patient with a periprosthetic joint infection (positive control), (B) a patient with a PG score of 2 (10–19 PG foci/10mm^2^), (C) a patient with a PG score of 3 (20–29 PG foci/10mm^2^), and (D) a patient with a PG score of 4 (30+ PG foci/10mm^2^). Enlarged insets show localization of PG foci (brown) within pockets of inflammation detectible by hematoxylin counterstain (blue).

To determine whether bacterial peptidoglycan was present in synovial tissue, we used immunohistochemistry (IHC) to stain for peptidoglycan (PG) in sections of fresh-frozen synovial tissue (Fig. 1). We validated our staining methodology using synovial tissue from 4 patients with periprosthetic joint infections to detect PG within the infected tissue (Fig. 1A). Using this validated method, we detected PG in 33/56 (59%) of synovial tissue from patients undergoing primary TKA with no history of joint infection. PG staining varied widely between patient samples, ranging from 0–94 PG occurrences per 10 mm^2^ of tissue.

**Figure 2:**
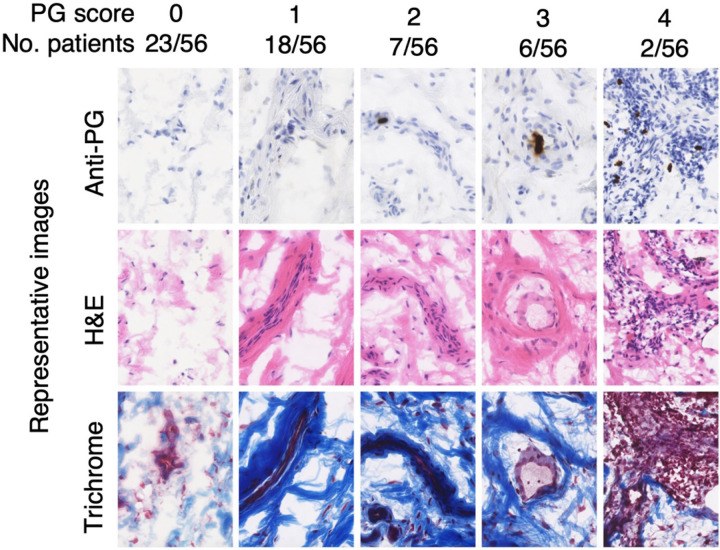
Association between peptidoglycan, synovial inflammation, and fibrosis. Shown are representative sections of synovial tissue stained for PG by immunohistochemistry (see methods for details). Inflammation and fibrosis were determined by H&E staining and Masson’s trichrome staining, respectively. Each column shows a representative sample that received a PG score 0–4 according to the quantity of PG staining foci per 10 mm^2^ (0=none, 1=1–9, 2=10–19, 3=20–29, 4=30+). Inflammatory infiltrate and localized areas of fibrosis frequently colocalized with the regions of high PG staining intensity.

Sample sections showed considerable variability in the degree of inflammation and fibrosis between patients, measured by H&E and trichrome staining, respectively (Fig. 2). PG in synovial tissue was typically localized within cells with both mononuclear and fibroblastic morphology (Supplemental Fig. S1), and these PG-positive cells were often surrounded by foci of inflammatory infiltrate and/or regions of fibrosis.

### Correlations between synovial tissue PG and clinical and laboratory findings

**Figure 3:**
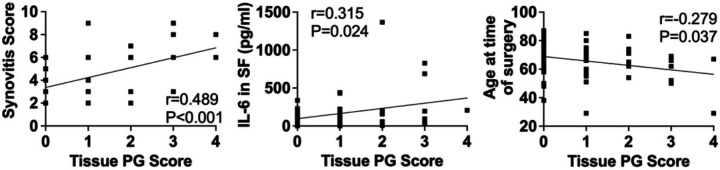
Correlations between synovial tissue PG score and clinical and laboratory findings. Pearson’s r were calculated to determine correlations between tissue PG score (0–4) and clinical and laboratory data. Shown are the correlation curves for correlations between PG score and synovitis severity, IL-6 in synovial fluid (SF), and age at the time of surgery. Calculated Pearson’s r and P values are indicated in the figure.

PG severity scores positively correlated with several clinical and laboratory findings (Fig. 3). Overall synovitis positively correlated with PG score (r = 0.489, p < 0.001). The level of IL-6 in synovial fluid also positively correlated with PG score (r = 0.315, p = 0.024). Additionally, there was a modest, significant inverse correlation between PG score and age at the time of surgery (r=−0.279, p = 0.037). Interestingly, there were no significant correlations between PG score and BMI, a well-studied risk factor for degenerative arthritis. Furthermore, there was no significant correlation between PG score and patient-reported outcomes.

### Inflammatory responses of synovial fibroblasts stimulated with PG

**Figure 4:**
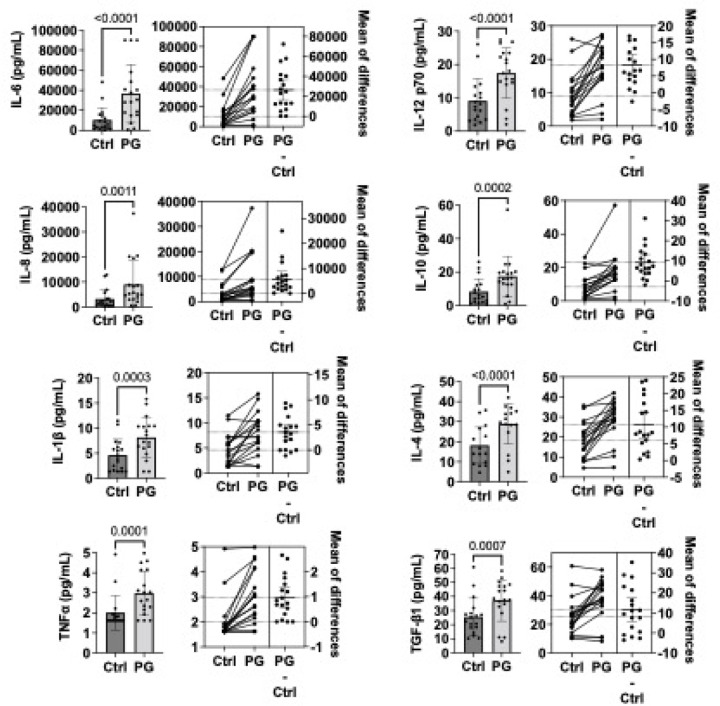
Cytokine secretion by primary human synovial fibroblasts stimulated with peptidoglycan (PG). Primary human synovial fibroblasts were isolated from 8 patients with osteoarthritis, 2 with joint trauma, 5 with Lyme arthritis, and 3 with rheumatoid arthritis, and passaged at least 6 times prior to stimulation. Cells were stimulated with 10 µg/ml of *S. aureus* PG muramyl dipeptide (Sigma-Aldrich) or media alone (ctrl) for 24 hours. Shown are mean (+/− SD) and estimation plots of pro-inflammatory (IL-6, IL-8, IL-1β, TNF, IL-12 p70) and anti-inflammatory/pro-fibrotic (IL-10, IL-4, TGF-β1) cytokines detected in cell culture supernatant by multiplex assay. Statistically significant differences between control and PG-stimulated cells were determined by paired two-tailed t test (p values and mean of differences are indicated in figure). Results stratified by disease type are available in supplemental material.

Synovial fibroblasts are the major cell type within the joint synovium. To determine the inflammatory responses of these tissue-resident cells, we isolated primary human synovial fibroblasts from 8 patients with osteoarthritis, collected as part of this study, as well as 5 with Lyme arthritis (LA) and 3 with rheumatoid arthritis (RA), collected previously. Cells were incubated in low-serum media and stimulated with the PG NOD2 ligand muramyl dipeptide from *S. aureus* for 24 hours. Cell supernatants were collected, and cytokines were analyzed by multiplex assay. PG-stimulated cells secreted elevated levels of numerous cytokines associated with inflammation (IL-1β, TNFα, IL-6, IL-8, IL-12p70) and tissue repair and fibrosis (IL-10, IL-4, TGF-β1). Of these cytokines, IL-6 levels were most significantly altered (p < 0.0001), with a ~ 4-fold increase in supernatants from PG-stimulated cells, compared with media alone controls (Fig. 4). These results were consistent with our *ex vivo* data (Fig. 3). Interestingly, results were similar across different types of synovial fibroblasts (OA vs. LA vs. RA, supplemental Fig. S2).

## Discussion

This study provides evidence that peptidoglycan (PG), a bacterial cell wall component, is present in the synovial tissue of over half of patients undergoing primary total knee arthroplasty for degenerative osteoarthritis. Furthermore, our results suggest that PG may play a role in the symptomatology and disease progression of osteoarthritis, as levels of PG positively correlated with synovitis and pro-inflammatory cytokine levels as well as younger age at time of arthroplasty. These results indicate that PG, likely derived from the microbiome, is involved in pathogenesis of knee osteoarthritis for at least a subset of patients with advanced knee OA. PG is a pathogen-associated molecular pattern (PAMP) that is recognized by several immune receptors that yield a pro-inflammatory response (18, 20, 21). Synovitis has been linked to clinical progression of OA (12, 13, 27, 28).

Historical paradigms held that the joint space was free of microbes and microbial debris in the absence of clinical infection, yet data has suggested that immune responses mediated by microbial byproducts may play a role in arthritis. The concept of microbial debris as a mediator of joint inflammation first emerged regarding inflammatory arthritis (24, 29–32). Newer data indicates that microbial debris, including PG as well as bacterial DNA fragments, is present in joint tissue in degenerative arthritis (17, 22, 23, 33). Supporting evidence for the role of microbial debris as a mediator of synovitis includes a study showing positive correlation between the PAMP lipopolysaccharide and knee OA severity (34). PG in particular has been shown in animal models to be strongly arthritogenic (17, 35, 36) and may be exploited as a potential therapeutic target (37). Our study is the first of this size to quantify PG in a cohort of patients with advanced knee OA and to characterize PG’s association with synovitis and inflammation. Together, our data and previous studies strongly support the premise that microbial debris derived from the host microbiome can act as a driver of synovitis in knee osteoarthritis.

There are several plausible mechanisms by which bacteria or bacterial byproducts from the host microbiome could travel to the knee joint hematogenously. PG has been identified in the blood of healthy individuals without clinical infection(38, 39). Potential sources of PG include gastrointestinal (GI), oral, and skin flora. Translocation of bacteria from the gastrointestinal tract through a permeable gut barrier has been postulated as a driver of surgical site infections (40); this phenomenon could also occur in the absence of clinical infection and could include bacterial byproducts. Boer et al found that gut dysbiosis is associated with joint pain and inflammation (41). Obesity, known to be strongly associated with OA, is linked to alterations in the gut microbiome that promotes increased absorption of bacterial byproducts (42, 43), although further studies are required to identify the source of PG in synovial tissue. Bacteria or bacterial byproducts may travel directly through the gut barrier, or could travel inside white blood cells (44, 45). Moentadj, *et al*, described the ability of PG-polysaccharide polymers from oral streptococcal species to induce arthritis in mice (35).

We found PG staining of synovium localized in cells with both macrophage and fibroblast morphologies. In seeming contradiction, Schrijver et al(24) previously showed localization of PG staining from synovial samples only within cells expressing markers of antigen presentation (HLA-DR, CD40, CD80, CD86) *in situ.* However, we and others have subsequently shown that synovia from Lyme arthritis(17) and rheumatoid arthritis(46) contain distinct populations of HLA-DR + synovial fibroblasts, particularly within the synovial sublining and perivascular regions. The cells with fibroblast morphology containing bacterial PG in this study display phenotypically similar characteristics. Our *in vitro* results further demonstrate that PG induces an inflammatory and fibrotic response in synovial fibroblasts, similar to senescent fibroblasts in other chronic inflammatory and fibrotic diseases(47). This is further supported by previous *ex-vivo* findings in PG-infected synovial cells(24). These data support a dual role for synovial fibroblasts, and likely other tissue-resident immune cells, as mediators of the pathogenic response to PG in synovium via upregulation of pro-inflammatory and pro-fibrotic cytokines.

We found no associations between PG and patient reported outcome measures following surgery. While the possibility of type II error cannot be excluded, we do not detect a strong signal that PG present at time of surgery is prohibitive of good outcome following knee replacement surgery. Nonetheless, further investigation of a possible role for PG to adversely affect post-TKA outcomes is warranted. There were no occurrences of periprosthetic joint infection in our elective TKA cohort out to 1 year following surgery. This indicates that the PG identified at time of surgery was not indicative of active clinical infection, but instead represented prior intrusion of these PAMPs into the joint space.

## Conclusions

In this study, we identified bacterial PG in patient synovium from over half (33/56) of patients with advanced knee OA undergoing arthroplasty. Furthermore, PG levels positively correlated with inflammatory markers, including inflammatory synovitis severity and elevated levels of IL-6 in synovial fluid. This *ex vivo* observation was supported by *in vitro* stimulation of primary human synovial fibroblasts with PG, which secreted high levels of both pro-inflammatory and pro-fibrotic cytokines, most notably IL-6. These findings implicate bacterial PG as an important contributor of joint inflammation and tissue damage in osteoarthritis. Further research is warranted to explore PG as a potential diagnostic and/or therapeutic target.

## Supplementary Material

Supplement 1

## Figures and Tables

**Table 1:** Patient Demographics and Patient Reported Outcomes

	Baseline Characteristics	3 months	6 months	12 months
**Sex**	31/56 female (55%)			
**Age** median (SD)	67 (12.8)			
**BMI** median (SD)	31.5 (5.9)			
**White**	41 (73%)			
**Black**	10 (18%)			
**Asian**	3 (5%)			
**Other**	2 (4%)			
**KOOS JR** mean (SD)	40.3 (15.5)	59.5 (10.8)	69.2 (13.1)	79.5 (16.1)
**PCS-12** mean (SD)	30.7 (9.6)	38.7 (9.4)	46.3 (12)	49.5 (12.7)
**MCS-12** mean (SD)	46.2 (6.1)	44.1 (5.7)	43.3 (4.5)	43.1 (3.7)

Baseline characteristics and outcomes at follow up are shown. SD, standard deviation; KOOS JR, Knee Injury and Osteoarthritis Outcome Score for Joint Replacement; PCS-12, Physical Component Score for VR-12 outcome; MCS-12, Mental Component Score for VR-12 outcome.

## Abbreviations

H&E: hematoxylin and eosin
IHC: immunohistochemistry
KOOS JR: Knee injury and Osteoarthritis Outcome Score for Joint Replacement
LA: Lyme arthritis
OA: osteoarthritis
PAMP: pathogen-associated molecular pattern
PG: peptidoglycan
RA: rheumatoid arthritis
TKA: total knee arthroplasty
VR-12: Veterans Rand-12 Health Survey

## References

[1] Dillon CF, Rasch EK, Gu Q, Prevalence of knee osteoarthritis in the United States: arthritis data from the Third National Health and Nutrition Examination Survey 1991–94. J Rheumatol. 2006;33(11):2271–9. 17013996

[2] Gill TM. Do the Tenets of Late-Life Disability Apply to Middle Age? Ann Intern Med. 2017;167(11):818–9. 29132158 10.7326/M17-2550 PMC5726542

[3] Hawker GA, Stewart L, French MR, Understanding the pain experience in hip and knee osteoarthritis--an OARSI/OMERACT initiative. Osteoarthritis Cartilage. 2008;16(4):415–22. 18296075 10.1016/j.joca.2007.12.017

[4] Bozic KJ, Rubash HE, Sculco TP, An analysis of medicare payment policy for total joint arthroplasty. J Arthroplasty. 2008;23(6 Suppl 1):133–8. 18555644 10.1016/j.arth.2008.04.013

[5] Shan L, Shan B, Suzuki A, Intermediate and long-term quality of life after total knee replacement: a systematic review and meta-analysis. J Bone Joint Surg Am. 2015;97(2):156–68. 25609443 10.2106/JBJS.M.00372

[6] Bourne RB, Chesworth BM, Davis AM, Patient satisfaction after total knee arthroplasty: who is satisfied and who is not? Clin Orthop Relat Res. 2010;468(1):57–63. 19844772 10.1007/s11999-009-1119-9 PMC2795819

[7] Beswick AD, Wylde V, Gooberman-Hill R, What proportion of patients report long-term pain after total hip or knee replacement for osteoarthritis? A systematic review of prospective studies in unselected patients. BMJ Open. 2012;2(1):e000435. 10.1136/bmjopen-2011-000435 PMC3289991 22357571

[8] Martel-Pelletier J, Barr AJ, Cicuttini FM, Osteoarthritis. Nat Rev Dis Primers. 2016;2:16072. 27734845 10.1038/nrdp.2016.72

[9] Loeser RF, Goldring SR, Scanzello CR, Osteoarthritis: a disease of the joint as an organ. Arthritis Rheum. 2012;64(6):1697–707. 22392533 10.1002/art.34453 PMC3366018

[10] de Lange-Brokaar BJ, Ioan-Facsinay A, van Osch GJ, Synovial inflammation, immune cells and their cytokines in osteoarthritis: a review. Osteoarthritis Cartilage. 2012;20(12):1484–99. 22960092 10.1016/j.joca.2012.08.027

[11] Ene R, Sinescu RD, Ene P, Synovial inflammation in patients with different stages of knee osteoarthritis. Rom J Morphol Embryol. 2015;56(1):169–73. 25826502

[12] Sellam J, Berenbaum F. The role of synovitis in pathophysiology and clinical symptoms of osteoarthritis. Nat Rev Rheumatol. 2010;6(11):625–35. 20924410 10.1038/nrrheum.2010.159

[13] Baker K, Grainger A, Niu J, Relation of synovitis to knee pain using contrast-enhanced MRIs. Ann Rheum Dis. 2010;69(10):1779–83. 20472593 10.1136/ard.2009.121426 PMC3885343

[14] Kapoor M, Martel-Pelletier J, Lajeunesse D, Role of proinflammatory cytokines in the pathophysiology of osteoarthritis. Nat Rev Rheumatol. 2011;7(1):33–42. 21119608 10.1038/nrrheum.2010.196

[15] Huang Z, Kraus VB. Does lipopolysaccharide-mediated inflammation have a role in OA? Nat Rev Rheumatol. 2016;12(2):123–9. 26656661 10.1038/nrrheum.2015.158 PMC4930555

[16] Loeser RF, Arbeeva L, Kelley K, Association of Increased Serum Lipopolysaccharide, But Not Microbial Dysbiosis, With Obesity-Related Osteoarthritis. Arthritis Rheumatol. 2022;74(2):227–36. 34423918 10.1002/art.41955 PMC8795472

[17] Jutras BL, Lochhead RB, Kloos ZA, Borrelia burgdorferi peptidoglycan is a persistent antigen in patients with Lyme arthritis. Proc Natl Acad Sci U S A. 2019;116(27):13498–507. 31209025 10.1073/pnas.1904170116 PMC6613144

[18] Wolf AJ, Underhill DM. Peptidoglycan recognition by the innate immune system. Nat Rev Immunol. 2018;18(4):243–54. 29292393 10.1038/nri.2017.136

[19] van der Heijden IM, Wilbrink B, Tchetverikov I, Presence of bacterial DNA and bacterial peptidoglycans in joints of patients with rheumatoid arthritis and other arthritides. Arthritis Rheum. 2000;43(3):593–8. 10728753 10.1002/1529-0131(200003)43:3<593::AID-ANR16>3.0.CO;2-1

[20] Boneca IG. The role of peptidoglycan in pathogenesis. Curr Opin Microbiol. 2005;8(1):46–53. 15694856 10.1016/j.mib.2004.12.008

[21] Royet J, Dziarski R. Peptidoglycan recognition proteins: pleiotropic sensors and effectors of antimicrobial defences. Nat Rev Microbiol. 2007;5(4):264–77. 17363965 10.1038/nrmicro1620

[22] Tarabichi M, Shohat N, Goswami K, Diagnosis of Periprosthetic Joint Infection: The Potential of Next-Generation Sequencing. J Bone Joint Surg Am. 2018;100(2):147–54. 29342065 10.2106/JBJS.17.00434

[23] Dunn CM, Velasco C, Rivas A, Identification of Cartilage Microbial DNA Signatures and Associations With Knee and Hip Osteoarthritis. Arthritis Rheumatol. 2020;72(7):1111–22. 31961065 10.1002/art.41210 PMC7336391

[24] Schrijver IA, Melief MJ, Tak PP, Antigen-presenting cells containing bacterial peptidoglycan in synovial tissues of rheumatoid arthritis patients coexpress costimulatory molecules and cytokines. Arthritis Rheum. 2000;43(10):2160–8. 11037875 10.1002/1529-0131(200010)43:10<2160::AID-ANR3>3.0.CO;2-T

[25] Jutras BL, Scott M, Parry B, Lyme disease and relapsing fever Borrelia elongate through zones of peptidoglycan synthesis that mark division sites of daughter cells. Proc Natl Acad Sci U S A. 2016;113(33):9162–70. 27506799 10.1073/pnas.1610805113 PMC4995948

[26] Brock AM, Jutras BL. A simple method to detect Borrelia burgdorferi sensu lato proteins in different sub-cellular compartments by immunofluorescence. Ticks Tick Borne Dis. 2021;12(6):101808. 34455142 10.1016/j.ttbdis.2021.101808

[27] Loeuille D, Chary-Valckenaere I, Champigneulle J, Macroscopic and microscopic features of synovial membrane inflammation in the osteoarthritic knee: correlating magnetic resonance imaging findings with disease severity. Arthritis Rheum. 2005;52(11):3492–501. 16255041 10.1002/art.21373

[28] Ayral X, Pickering EH, Woodworth TG, Synovitis: a potential predictive factor of structural progression of medial tibiofemoral knee osteoarthritis -- results of a 1 year longitudinal arthroscopic study in 422 patients. Osteoarthritis Cartilage. 2005;13(5):361–7. 15882559 10.1016/j.joca.2005.01.005

[29] Lichtman SN, Bachmann S, Munoz SR, Bacterial cell wall polymers (peptidoglycan-polysaccharide) cause reactivation of arthritis. Infect Immun. 1993;61(11):4645–53. 8406862 10.1128/iai.61.11.4645-4653.1993 PMC281216

[30] Melief MJ, Hoijer MA, Van Paassen HC, Presence of bacterial flora-derived antigen in synovial tissue macrophages and dendritic cells. Br J Rheumatol. 1995;34(12):1112–6. 8608350 10.1093/rheumatology/34.12.1112

[31] Schrijver IA, Melief MJ, Markusse HM, Peptidoglycan from sterile human spleen induces T-cell proliferation and inflammatory mediators in rheumatoid arthritis patients and healthy subjects. Rheumatology (Oxford). 2001;40(4):438–46. 11312384 10.1093/rheumatology/40.4.438

[32] Hadler NM, Granovetter DA. Phlogistic properties of bacterial debris. Semin Arthritis Rheum. 1978;8(1):1–16. 358396 10.1016/0049-0172(78)90031-8

[33] Zhao Y, Chen B, Li S, Detection and characterization of bacterial nucleic acids in culture-negative synovial tissue and fluid samples from rheumatoid arthritis or osteoarthritis patients. Sci Rep. 2018;8(1):14305. 30250232 10.1038/s41598-018-32675-w PMC6155189

[34] Huang ZY, Stabler T, Pei FX, Both systemic and local lipopolysaccharide (LPS) burden are associated with knee OA severity and inflammation. Osteoarthritis Cartilage. 2016;24(10):1769–75. 27216281 10.1016/j.joca.2016.05.008 PMC5026878

[35] Moentadj R, Wang Y, Bowerman K, Streptococcus species enriched in the oral cavity of patients with RA are a source of peptidoglycan-polysaccharide polymers that can induce arthritis in mice. Ann Rheum Dis. 2021;80(5):573–81. 33397732 10.1136/annrheumdis-2020-219009

[36] Wu HJ, Ivanov II, Darce J, Gut-residing segmented filamentous bacteria drive autoimmune arthritis via T helper 17 cells. Immunity. 2010;32(6):815–27. 20620945 10.1016/j.immuni.2010.06.001 PMC2904693

[37] Huang Z, Wang J, Xu X, Antibody neutralization of microbiota-derived circulating peptidoglycan dampens inflammation and ameliorates autoimmunity. Nat Microbiol. 2019;4(5):766–73. 30833732 10.1038/s41564-019-0381-1

[38] Alexander KL, Targan SR, Elson CO 3rd. Microbiota activation and regulation of innate and adaptive immunity. Immunol Rev. 2014;260(1):206–20. 24942691 10.1111/imr.12180 PMC4080089

[39] Xu XL, Lee RT, Fang HM, Bacterial peptidoglycan triggers Candida albicans hyphal growth by directly activating the adenylyl cyclase Cyr1p. Cell Host Microbe. 2008;4(1):28–39. 18621008 10.1016/j.chom.2008.05.014

[40] Alverdy JC, Hyman N, Gilbert J. Re-examining causes of surgical site infections following elective surgery in the era of asepsis. Lancet Infect Dis. 2020;20(3):e38–e43. 10.1016/S1473-3099(19)30756-X PMC8019154 32006469

[41] Boer CG, Radjabzadeh D, Medina-Gomez C, Intestinal microbiome composition and its relation to joint pain and inflammation. Nat Commun. 2019;10(1):4881. 31653850 10.1038/s41467-019-12873-4 PMC6814863

[42] Luck H, Tsai S, Chung J, Regulation of obesity-related insulin resistance with gut anti-inflammatory agents. Cell Metab. 2015;21(4):527–42. 25863246 10.1016/j.cmet.2015.03.001

[43] Brun P, Castagliuolo I, Di Leo V, Increased intestinal permeability in obese mice: new evidence in the pathogenesis of nonalcoholic steatohepatitis. Am J Physiol Gastrointest Liver Physiol. 2007;292(2):G518–25. 17023554 10.1152/ajpgi.00024.2006

[44] Thwaites GE, Gant V. Are bloodstream leukocytes Trojan Horses for the metastasis of Staphylococcus aureus? Nat Rev Microbiol. 2011;9(3):215–22. 21297670 10.1038/nrmicro2508

[45] Chisari E, Cho J, Wouthuyzen-Bakker M, Periprosthetic Joint Infection and the Trojan Horse Theory: Examining the Role of Gut Dysbiosis and Epithelial Integrity. J Arthroplasty. 2022;37(7):1369–74. 35301048 10.1016/j.arth.2022.03.030

[46] Mizoguchi F, Slowikowski K, Wei K, Functionally distinct disease-associated fibroblast subsets in rheumatoid arthritis. Nat Commun. 2018;9(1):789. 29476097 10.1038/s41467-018-02892-y PMC5824882

[47] He S, Sharpless NE. Senescence in Health and Disease. Cell. 2017;169(6):1000–11. 28575665 10.1016/j.cell.2017.05.015 PMC5643029

