# Supplementary material for: Peptidoglycan in osteoarthritis synovial tissue is associated with joint inflammation
Source: Res Sq. 2023 Apr 28:rs.3.rs-2842385. Preprint. [Version 1] doi: 10.21203/rs.3.rs-2842385/v1 (PMC10168439; doi:10.21203/rs.3.rs-2842385/v1)
Supplement: Supplement 1 [file NIHPPRS2842385V1-supplement-1.pdf]

Supplementary Files

This is a list of supplementary files associated with this preprint. Click to download.

- FigS1wLegend.jpg
- FigS2wlegend.jpg
